# M2 macrophage-related molecular subtypes and prognostic index for prostate cancer patients through integrating single-cell and bulk RNA sequencing analysis

**DOI:** 10.1016/j.gendis.2023.101086

**Published:** 2023-09-09

**Authors:** Dechao Feng, Xu Shi, Dengxiong Li, Ruicheng Wu, Jie Wang, Wuran Wei, Ping Han

**Affiliations:** Department of Urology, Institute of Urology, West China Hospital, Sichuan University, Chengdu, Sichuan 610041, China

Prostate cancer (PCa) is considered as an age-related disease and accounts for the most prevalence of urinary malignancies in men.^1^ We previously analyzed the cellular landscape of tumor microenvironment in PCa patients, where we found that cancer-related fibroblasts might play an important role in tumorigenesis and the development of PCa and tumor-associated macrophage (TAM) could function synergistically with them.^2^ Thus, we further integrated single-cell and bulk RNA sequencing with meta-analysis to specify the prognostic effect of TAM on PCa and construct molecular subgroups and predictive index to guide clinical practice.

We perceived that M2 macrophage was substantially linked with biochemical recurrence (BCR)-free survival in 430 PCa patients in the TCGA database using the Cibersortx algorithm (Fig. 1A). Subsequently, we downloaded 271 markers related to TAM from the tumor immunotherapy gene expression resource (TIGER) database.^3^ Using Pearson analysis, 92 genes from the TCGA database were connected to the M2 macrophage, and 14 genes obtained from the intersection of M2 macrophage-related genes and TAM markers (Fig. 1B) were entered into the Lasso regression analysis. When lambda (λ) equaled 0.0123 (Fig. 1C), we obtained the optimal model and determined FCGR2A, APOE, and MS4A7 as final genes (Fig. 1D). The three genes could clearly classify the PCa patients into two subtypes in the TCGA database (Fig. 1E), as well as other two independent cohorts. Meta-analysis results indicated that the BCR risk of subtype 2 was 1.82 times higher than that of subtype 1 with statistical significance (Fig. 1F). To better guide clinical practice, we created a risk score based on the aforementioned genes and split the 430 PCa patients in the TCGA database into high- and low-risk groups based on the risk score's median value. We found that the high-risk group had a significantly higher risk of BCR than the low-risk group (HR: 1.94; Fig. 1G). The other three cohorts showed similar results, and meta-analysis showed that the high-risk group had twice the risk of BCR than the low-risk group (Fig. 1G).

**Figure 1:**
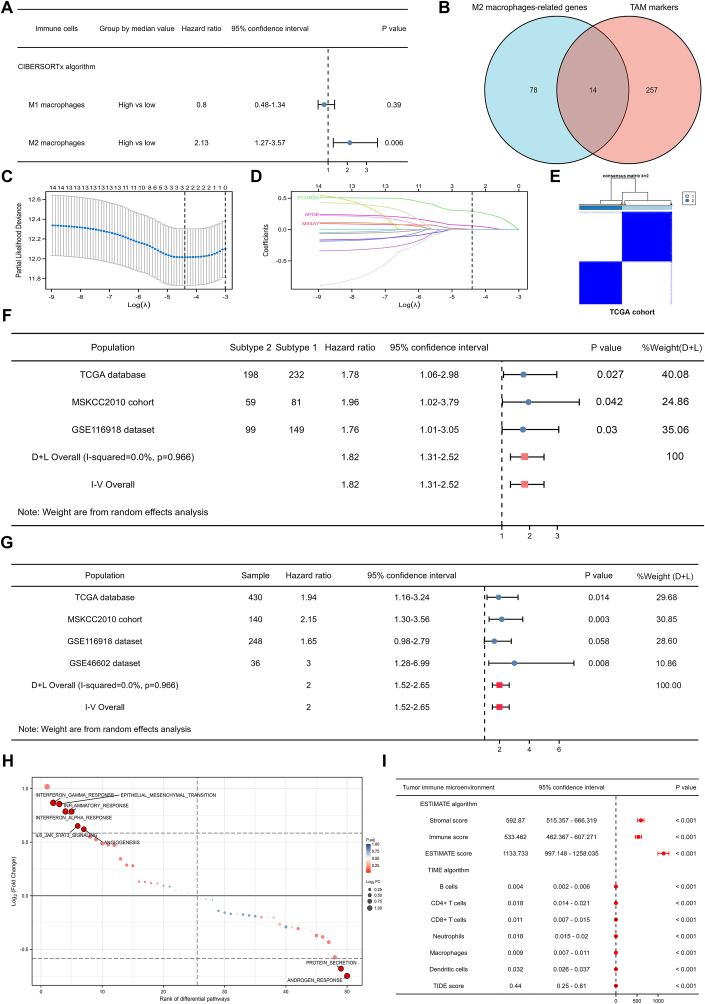
M2 macrophage-related molecular subtypes and prognostic index for prostate cancer patients. **(A)** The forest plot illustrating the impact of M1 and M2 macrophages on prostate cancer survival. **(B)** The Venn plot illustrating the intersection between TAM-related genes and markers. **(C)** The ideal model illustrated by a Lasso regression analysis. **(D)** The identified three genes shown by a Lasso regression analysis. **(E)** Two distinct molecular subtypes in the TCGA database using the above three genes. **(F)** The forest plot showing the meta-analysis results of biochemical recurrence-free survival differences of two molecular subtypes in three cohorts. **(G)** The forest plot displaying the meta-analysis results of biochemical recurrence-free survival differences of high- and low-risk groups in four cohorts. **(H)** The rank of differential pathways using gene set variation analysis. **(I)** The forest plot showing tumor immune microenvironment and checkpoints differences of two subtypes in the TCGA database. TAM, tumor-associated macrophage.

In addition, the baseline comparison showed that the patients in subtype 2 were older and had higher Gleason scores, and more of the patients tested positive for residual tumors (Table S1). The top five genes between subtype 2 and subtype 1 were *KMT2D*, *KDM6A*, *CACNA1A*, *SACS*, and *SCN10A* with statistical significance (Fig. S3E). For functional analysis, interferon-gamma response, epithelial–mesenchymal transition, inflammatory response, interferon alpha response, IL6-JAK-STAT3 signaling, and angiogenesis were highly enriched in subtype 2 while androgen response and protein secretion were highly enriched in subtype 1 (Fig. 1H). The expression levels of most checkpoints were significantly higher in subtype 2 than those in subtype 1 (Fig. S4B). In addition, subtype 2 had significantly higher TME scores and immune cell scores than subtype 1, as well as TIDE scores (Fig. 1I). In terms of tumor heterogeneity and stemness, subtype 2 showed higher levels of HRD, LOH, TMB, DMPss, DNAss, and ENHss than subtype 1 but had lower levels of purity, mRNAsi, and RNAss than subtype 1 (Fig. S4D). The full-length article and detailed methods could be seen in the supplementary material.

TAM makes up the majority of innate immune cells and 50% of the mass of human tumor cells. Our findings have been corroborated in part by earlier research,^4^ which demonstrates that the majority of PCa stromal cells have the M2 phenotype and that a higher prevalence of M2 is associated with a worse prognosis, a faster rate of cancer progression, more frequent tumor extension, a higher BCR, and a higher stage and Gleason stage following radical prostatectomy. There are no known tissue-specific macrophage population markers in PCa. Therefore, using a classification system based on FCGR2A, APOE, and MS4A7, we tried to categorize macrophage subtypes at the gene level, a more upstream deciding factor for markers. Additionally, we note that subtype 2 had much higher TME and immune cell ratings than subtype 1, which suggests that M2 macrophages and tumor stroma may interact extensively. The PCa microenvironment is made up of an enrichment of active CAFs and M2-like macrophages. These stromal elements actively encourage increased aggressiveness in PCa cells, which ultimately helps cancer cells escape from the original tumor and spread metastatically. Interestingly, we discovered that the low-risk group had higher levels of both protein synthesis and androgen responses. This may be connected to the idea that androgens have anti-inflammatory qualities, blocking pro-inflammatory reactions triggered by estrogen/ER through the androgen receptor. Prostate cancer cell lines are encouraged to migrate and invade by androgen receptor activation in macrophages.^5^

This study also has significant drawbacks, including the fact that blood-derived macrophages and embryo-derived macrophages both make up prostate macrophages. Additionally, PCa should highlight compartment-specific macrophages because their geographically diverse distribution can produce contradictory outcomes when TAMs are used to predict illness.

To the best of the authors' knowledge, our study is the first to show how single-cell and bulk RNA sequencing, as well as meta-analysis, may effectively combine to predict the occurrence of BCR in PCa patients using M2 macrophage-related prognostic markers. This shows that the immunological microenvironment and the androgen response in prostate cancer are strongly connected. One of the study avenues could be to develop future medications that specifically target particular TAM subtypes. Overall, we found M2 macrophage-related prognostic index and two distinct molecular subtypes closely associated with androgen response and immune microenvironment in prostate cancer which might be important for future research of this disease.

## Ethics declaration

The authors are accountable for all aspects of the work in ensuring that questions related to the accuracy or integrity of any part of the work are appropriately investigated and resolved.

## Data availability

The results shown here are in whole or part based upon data generated by the TCGA Research Network: https://www.cancer.gov/tcga.

## Conflict of interests

The authors declare that there is no conflict of interests.
