# Supplementary material for: M2 macrophage-related molecular subtypes and prognostic index for prostate cancer patients through integrating single-cell and bulk RNA sequencing analysis
Source: Genes Dis. 2023 Sep 9;11(4):101086. doi: 10.1016/j.gendis.2023.101086 (PMC10955206; doi:10.1016/j.gendis.2023.101086)
Supplement: Multimedia component 1 [file mmc1.docx]

**Methods**

**Data preparation**

We downloaded 271 markers related to TAM from the tumor immunotherapy gene expression resource (TIGER) database (<http://tiger.canceromics.org/#/singleCellImmune>) (1), which contains single-cell transcriptome data of 2,116,945 immune cells from 655 samples including prostate cancer (2). We used the PCa gene matrix and clinical features in TCGA database from our pervious study (3). TAM abundance, including M1 and M2, was calculated using Cibersortx algorithm (4), and prognosis analysis was conducted. The M2 macrophage-related genes were calculated using a Pearson analysis, and the requirements were that the P value be less than 0.5 and the absolute value of the coefficient be greater than 0.3. After TAM markers and genes associated with M2 macrophages intersected, we used Lasso regression analysis to identify the remaining genes. Then, based on these genes, we created a risk score using the coefficients from the Lasso regression and the TCGA subtypes. Risk score = 0.290751140810346 * FCGR2A + 0.000967447558905841 *MS4A7 + 0.0587206755698891*APOE. 430 samples from the TCGA database were examined, and the log-rank test for BCR-free survival yielded a p value less than 0.05. Three cohorts were used externally validated the prognostic values of risk score and TCGA subtypes, including GSE46602 (5), GSE116918 (6) and MSKCC2010 (7, 8). Analysis was done on the clinical characteristics of molecular subtypes. Additionally, we combined the aforementioned data of the risk score and molecular subtypes using Stata 14.0 software.

**Mutaion landscape and functional diferences between two subtypes**

The TCGA database (https://portal.gdc.com), which contains information on PCa, was used to download RNA-sequencing profiles, genetic mutations, and related clinical data.The data of mutations were downloaded and visualized using the maftools package in R software. A comparison of the differences in mutation frequency between the two kinds was also done. In terms of functional analysis, "h.all.v7.4.symbols.gmt" from the molecular signatures database was used to undertake gene set variation analysis (9, 10). The number of genes in the set ranged from 5 to 5000. The "wilcox.test" programme was then used to assess how each pathway differed between the two clusters. The fold change was 1.5, and we defined statistical significance as p. adj. 0.01 and false discovery rate 0.01 respectively.

**Tumor stemness and heterogeneity analyses**

Tumor stemness indexes contained differentially methylated probes-based stemness scores (DMPss), DNA methylation-based stemness scores (DNAss), enhancer elements/DNA methylation-based stemness scores (ENHss), epigenetically regulated DNA methylation-based stemness scores (EREG-METHss), epigenetically regulated RNA expression-based stemness scores (EREG.EXPss), RNA expression-based stemness scores (RNAss) (11) and mRNAsi algorithm (12). Loss of heterozygosity (LOH), neoantigen (NEO), tumour ploidy, tumour purity, mutant-allele tumour heterogeneity (MATH), tumour mutation burden (TMB), and microsatellite instability (MSI) were all markers of tumour heterogeneity (13, 14). The results of above indicators were obtained from our previous study (15). We used the Wilcoxon rank sum test to compare the differences between two subtypes.

**TME evaluation**

The TIMER and ESTIMATE algorithms assessed the entire tumour microenvironment and immunological components (16-18). With the help of the tumour immune dysfunction and exclusion (TIDE) algorithm, the potential response to immune checkpoint blockade (ICB) therapy was predicted (19). High TIDE scores are correlated with poor ICB efficacy. The Wilcoxon rank sum test was used to compare the differences in 54 immunological checkpoints and tumour microenvironment scores between the two subtypes. Supplementary Figure 1 illustrates the study flowchart.

**Statistical analysis**

We performed the analysis using R 3.6.3 and the appropriate tools. In the case of an anomalous distribution, we took advantage of the Wilcoxon test. A Kaplan-Meier curve representing the results of the log-rank test was used for the survival analysis. The threshold for statistical significance was two-sided p 0.05. Not significance (ns), p≥0.05; *, p<0.05; **, p<0.01; ***, p<0.001 were the significant markings.

**References**

1. Chen Z, Luo Z, Zhang D, Li H, Liu X, Zhu K, et al. TIGER: A Web Portal of Tumor Immunotherapy Gene Expression Resource. Genomics Proteomics Bioinformatics. 2022.

2. Chen S, Zhu G, Yang Y, Wang F, Xiao YT, Zhang N, et al. Single-cell analysis reveals transcriptomic remodellings in distinct cell types that contribute to human prostate cancer progression. Nat Cell Biol. 2021;23(1):87-98.

3. Feng D, Shi X, Zhang F, Xiong Q, Wei Q, Yang L. Energy Metabolism-Related Gene Prognostic Index Predicts Biochemical Recurrence for Patients With Prostate Cancer Undergoing Radical Prostatectomy. Front Immunol. 2022;13:839362.

4. Newman AM, Steen CB, Liu CL, Gentles AJ, Chaudhuri AA, Scherer F, et al. Determining cell type abundance and expression from bulk tissues with digital cytometry. Nat Biotechnol. 2019;37(7):773-82.

5. Mortensen MM, Hoyer S, Lynnerup AS, Orntoft TF, Sorensen KD, Borre M, et al. Expression profiling of prostate cancer tissue delineates genes associated with recurrence after prostatectomy. Sci Rep. 2015;5:16018.

6. Jain S, Lyons CA, Walker SM, McQuaid S, Hynes SO, Mitchell DM, et al. Validation of a Metastatic Assay using biopsies to improve risk stratification in patients with prostate cancer treated with radical radiation therapy. Ann Oncol. 2018;29(1):215-22.

7. Cerami E, Gao J, Dogrusoz U, Gross BE, Sumer SO, Aksoy BA, et al. The cBio cancer genomics portal: an open platform for exploring multidimensional cancer genomics data. Cancer Discov. 2012;2(5):401-4.

8. Gao J, Aksoy BA, Dogrusoz U, Dresdner G, Gross B, Sumer SO, et al. Integrative analysis of complex cancer genomics and clinical profiles using the cBioPortal. Sci Signal. 2013;6(269):pl1.

9. Liberzon A, Subramanian A, Pinchback R, Thorvaldsdottir H, Tamayo P, Mesirov JP. Molecular signatures database (MSigDB) 3.0. Bioinformatics. 2011;27(12):1739-40.

10. Hanzelmann S, Castelo R, Guinney J. GSVA: gene set variation analysis for microarray and RNA-seq data. BMC Bioinformatics. 2013;14:7.

11. Huang TX, Fu L. The immune landscape of esophageal cancer. Cancer Commun (Lond). 2019;39(1):79.

12. Malta TM, Sokolov A, Gentles AJ, Burzykowski T, Poisson L, Weinstein JN, et al. Machine Learning Identifies Stemness Features Associated with Oncogenic Dedifferentiation. Cell. 2018;173(2):338-54 e15.

13. Bonneville R, Krook MA, Kautto EA, Miya J, Wing MR, Chen HZ, et al. Landscape of Microsatellite Instability Across 39 Cancer Types. JCO Precis Oncol. 2017;2017.

14. Thorsson V, Gibbs DL, Brown SD, Wolf D, Bortone DS, Ou Yang TH, et al. The Immune Landscape of Cancer. Immunity. 2018;48(4):812-30 e14.

15. Feng Dechao SX, Zhu Weizhen, Zhang Facai, Li Dengxiong, Han Ping, Wei Qiang, Yang Lu. . A pan-cancer analysis of the oncogenic role of leucine zipper protein 2 in human cancer. Exp Hematol Oncol 2022;11(1):55. (1):55.

16. Li B, Severson E, Pignon JC, Zhao H, Li T, Novak J, et al. Comprehensive analyses of tumor immunity: implications for cancer immunotherapy. Genome Biol. 2016;17(1):174.

17. Yoshihara K, Shahmoradgoli M, Martinez E, Vegesna R, Kim H, Torres-Garcia W, et al. Inferring tumour purity and stromal and immune cell admixture from expression data. Nat Commun. 2013;4:2612.

18. Zeng D, Ye Z, Shen R, Yu G, Wu J, Xiong Y, et al. IOBR: Multi-Omics Immuno-Oncology Biological Research to Decode Tumor Microenvironment and Signatures. Front Immunol. 2021;12:687975.

19. Jiang P, Gu S, Pan D, Fu J, Sahu A, Hu X, et al. Signatures of T cell dysfunction and exclusion predict cancer immunotherapy response. Nat Med. 2018;24(10):1550-8.

| Characteristic | Subtype 1 | Subtype 2 | P value |
| --- | --- | --- | --- |
| Sample | 232 | 198 |  |
| Age, median (IQR) | 60 (55, 66) | 63 (58, 66) | 0.004 |
| Gleason score, n (%) |  |  | < 0.001 |
| 6 | 25 (5.8%) | 14 (3.3%) |  |
| 7 | 134 (31.2%) | 72 (16.7%) |  |
| 8 | 30 (7%) | 29 (6.7%) |  |
| 9 | 43 (10%) | 83 (19.3%) |  |
| T stage, n (%) |  |  | 0.119 |
| T2 | 93 (21.9%) | 62 (14.6%) |  |
| T3 | 133 (31.4%) | 128 (30.2%) |  |
| T4 | 3 (0.7%) | 5 (1.2%) |  |
| Race, n (%) |  |  | 0.430 |
| ASIAN | 8 (1.9%) | 3 (0.7%) |  |
| BLACK OR AFRICAN AMERICAN | 26 (6.2%) | 24 (5.8%) |  |
| WHITE | 189 (45.4%) | 166 (39.9%) |  |
| N stage, n (%) |  |  | 0.064 |
| N0 | 169 (45.1%) | 137 (36.5%) |  |
| N1 | 29 (7.7%) | 40 (10.7%) |  |
| Residual tumor, n (%) |  |  | < 0.001 |
| No | 164 (39.1%) | 109 (26%) |  |
| Yes | 62 (14.8%) | 84 (20%) |  |

**Supplementary Table 1.** The clinical differences between two TCGA database.

IQR: interquartile range.

**Supplementary Figure 1.** The flowchart of this study.


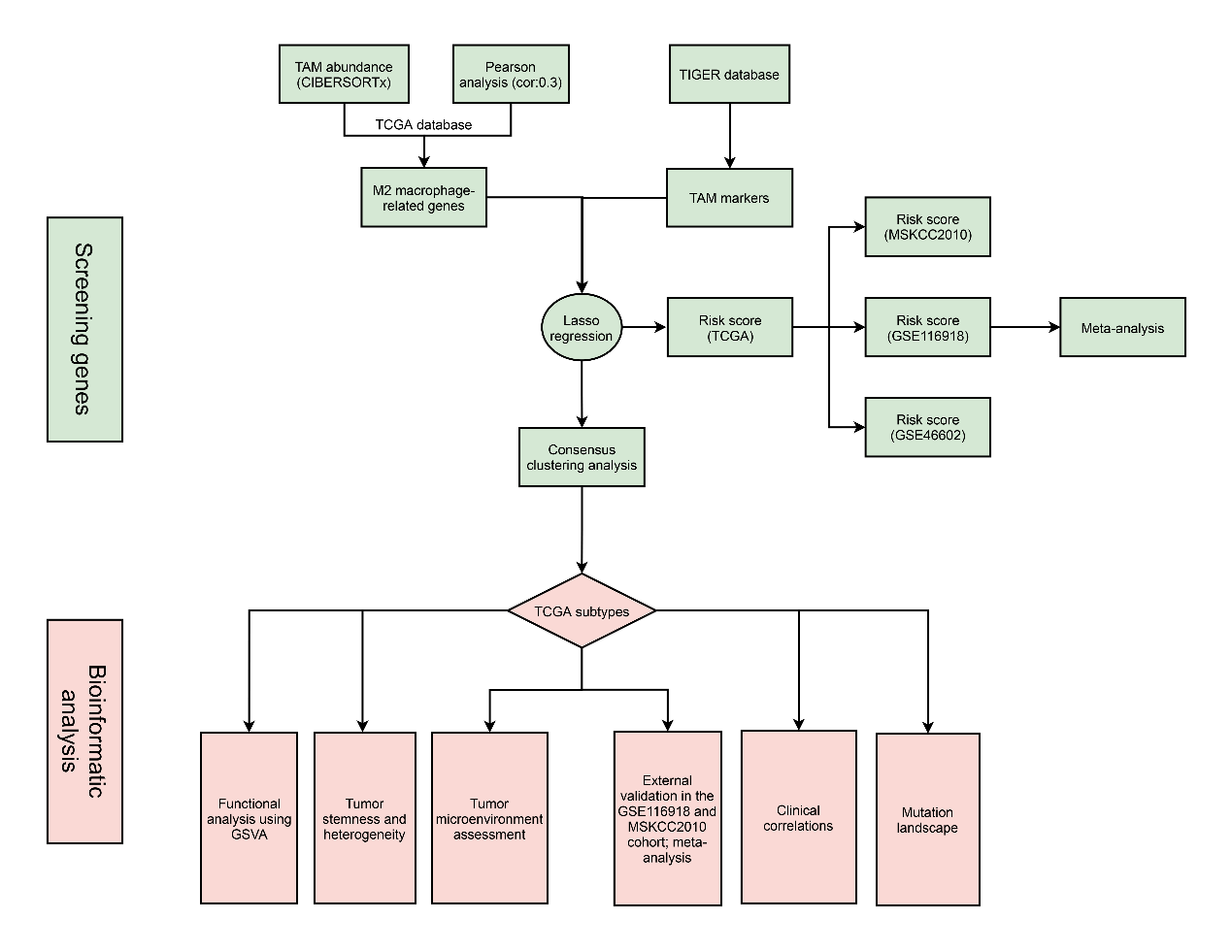


**Supplementary Figure 2.** Identifying TAM-related markers and constructing gene prognostic index
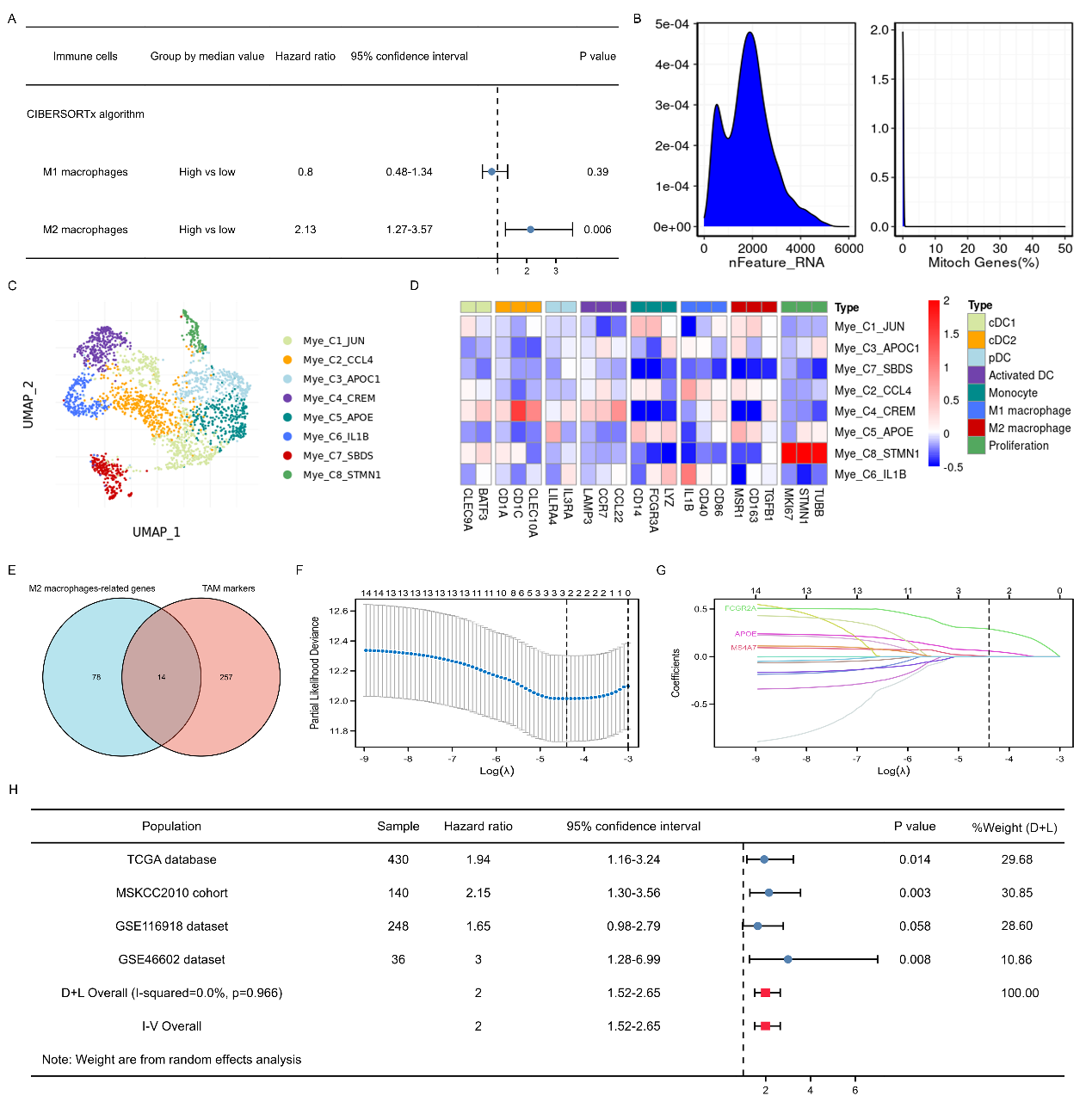
(A) forest plot showing the effect of M1 and M2 macrophage on prostate cancer survival; (B) quality control of single-cell analysis; (C) cell cluster of single-cell analysis; (C) cell makers of single-cell analysis; (D) Venn plot showing intersection of TAM-related genes and markers; (F) Lasso regression analysis showing the optimal model; (G) Lasso regression analysis showing the selected genes; (H) forest plot showing the meta-analysis results of biochemical recurrence-free survival differences of high- and low-risk groups in four cohorts; prostate cancer patients were divided into high- and low-risk groups according to the median value of gene prognositc index based on the above selected three genes. TAM= tumor-associated macrophage.

**Supplementary Figure 3.** Identification of M2 macrophage-related molecular subtypes and mutation analysis between two subtypes in TCGA database.


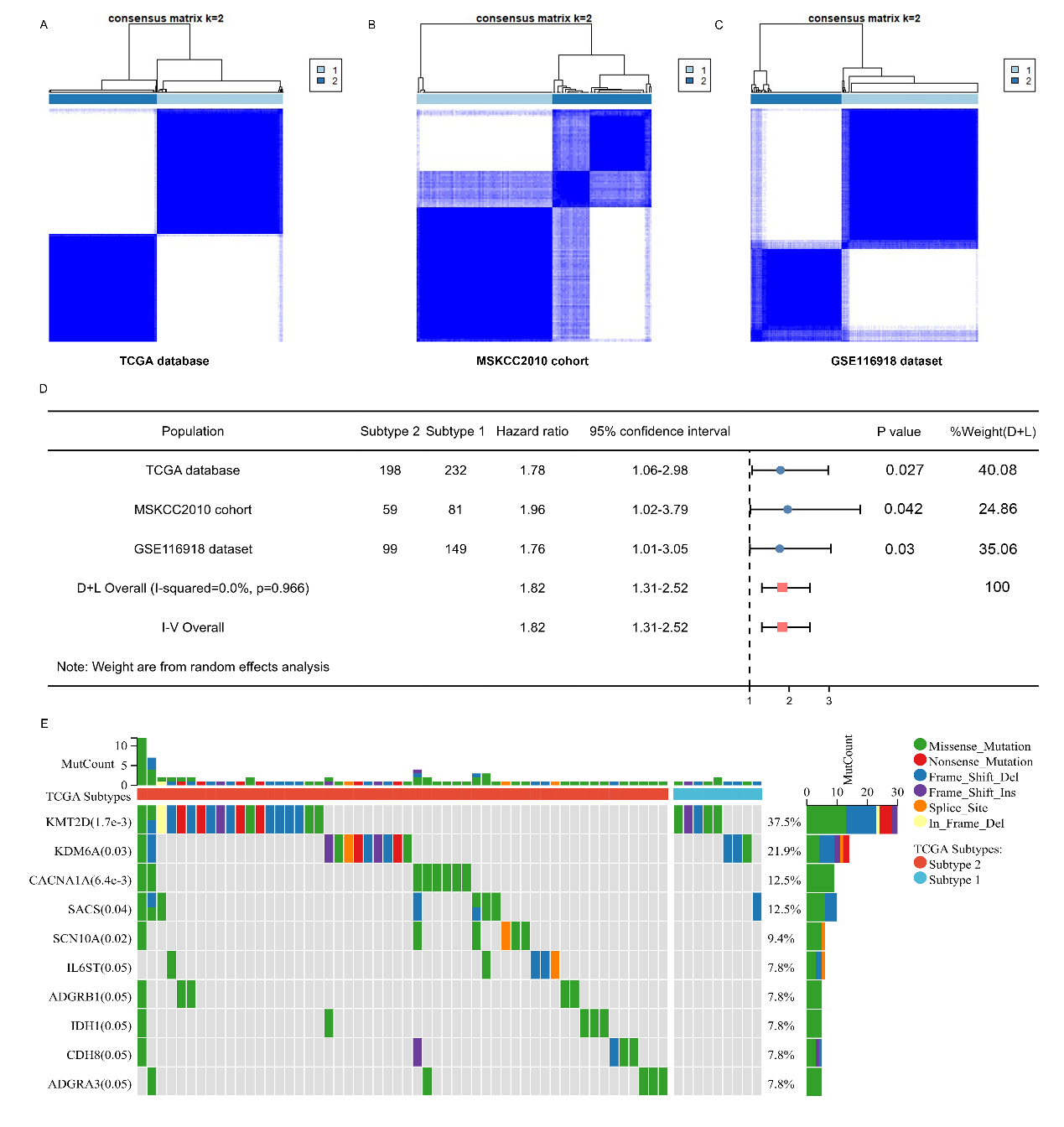


(A) two distinct molecular subtypes in TCGA database; (B) two distinct molecular subtypes in MSKCC2010 cohort; (C) two distinct molecular subtypes in GSE116918 cohort; (D) forest plot showing the meta-analysis results of biochemical recurrence-free survival differences of two molecular subtypes in three cohorts; (E) Waterfall plot showing the top ten mutation gene analysis between two subtypes in TCGA database.

**Supplementary Figure 4.** Gene set variation analysis, immune-related assessment and comparison of tumor heterogeneity and stemness .


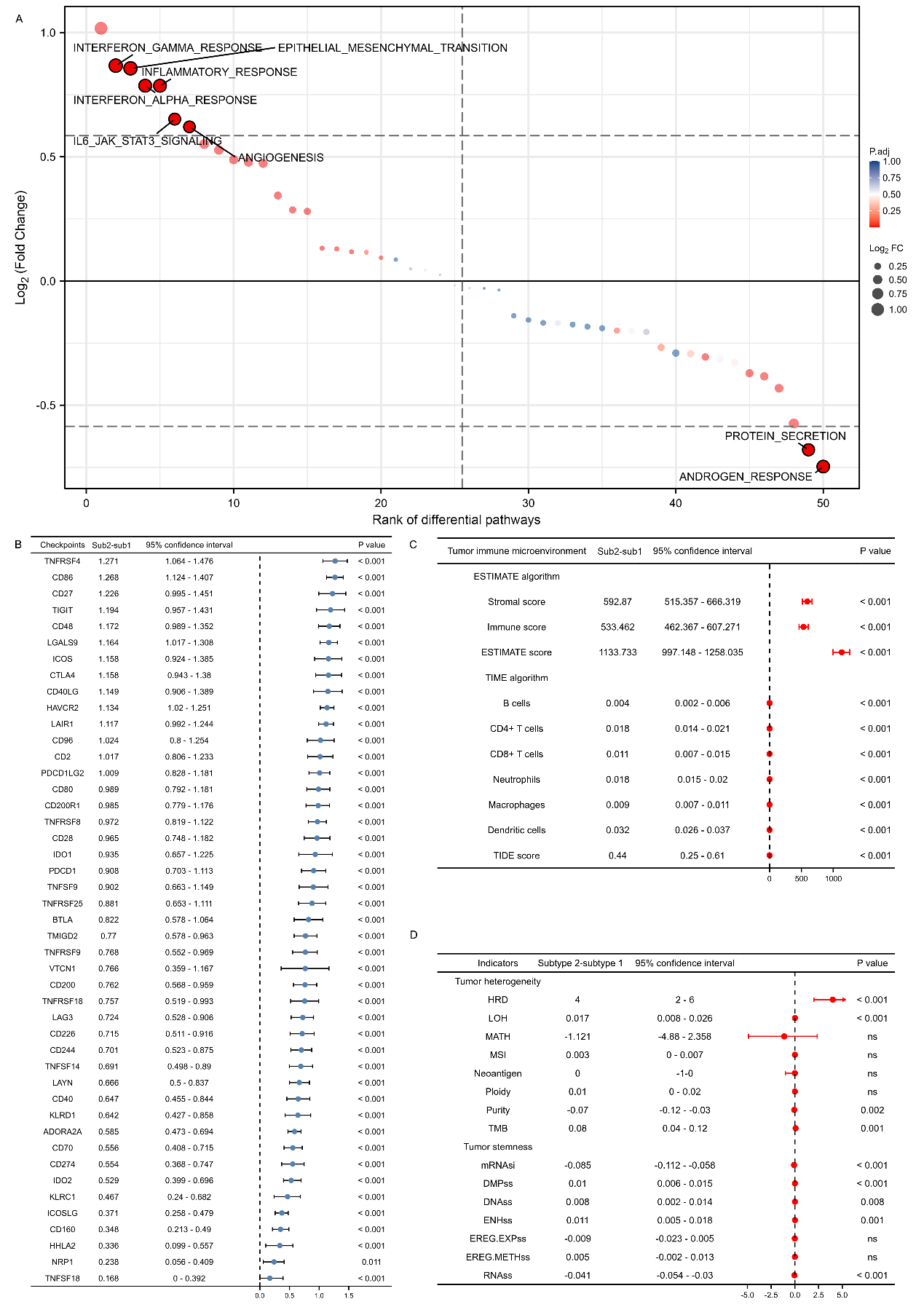


(A) rank of differential pathways using gene set variation analysis; (B) forest plot showing significant differences of checkpoints between two subtypes in TCGA database; (C) forest plot showing tumor immune microenvironment and checkpoints differences of two subtypes in TCGA database; (D) forest plot showing tumor heterogeneity and stemness differences of two subtypes in TCGA database. TIME= tumor immune microenvironment; DMPss=differentially methylated probes-based stemness scores; DNAss=DNA methylation-based stemness scores; ENHss=enhancer elements/DNA methylation-based stemness scores; EREG-METHss=epigenetically regulated DNA methylation-based stemness scores; EREG.EXPss=epigenetically regulated RNA expression-based stemness scores; RNAss=RNA expression-based stemness scores; HRD=homologous recombination deficiency; LOH=loss of heterozygosity; MATH=mutant-allele tumor heterogeneity; TMB=tumor mutation burden; MSI=microsatellite instability; TIDE=tumor immune dysfunction and exclusion.
